# Echocardiography guided bed side balloon atrial septostomy in dextro transposed great arteries (dTGA) with intact ventricular septum (IVS): A resource limited country experience

**DOI:** 10.12669/pjms.346.15792

**Published:** 2018

**Authors:** Naresh Kumar, Abdul Sattar Shaikh, Veena Kumari, Najma Patel

**Affiliations:** 1 *Naresh Kumar, FCPS, Fellow Paediatric Cardiology, NICVD Karachi, Pakistan*; 2 *Abdul Sattar Shaikh, FCPS, Assistant Professor of Paediatric Cardiology, NICVD Karachi, Pakistan*; 3 *Veena Kumari, FCPS, Fellow Paediatric Cardiology, NICVD Karachi, Pakistan*; 4 *Najma Patel, FCPS, FSCAI, Professor and Head of the Department, Paediatric Cardiology, NICVD Karachi, Pakistan*

**Keywords:** Congenital heart diseases (CHD), dextro Transposition of Great Arteries (d-TGA), Intact Ventricular Septum (IVS), Balloon Atrial Septostomy (BAS), Patent Foramen Ovale (PFO), Atrial Septal Defect (ASD)

## Abstract

**Objective:**

To determine effectiveness and safety of echocardiography guided bed side Balloon Atrial Septostomy (BAS) in dextro transposition of great Arteries (dTGA) with intact ventricular septum (IVS) at a public sector tertiary care hospital Karachi, Pakistan.

**Methods:**

This case series include 40 patients with echocardiographic findings of dTGA with IVS and restricted PFO (≤ 2mm) who underwent bedside BAS at department of pediatric cardiology NICVD, Karachi, Pakistan. We recorded pre and post BAS diameter of PFO/Atrial Septal Defect (ASD), oxygen saturation (SpO_2_ %), and post procedure complications and outcome.

**Results:**

Median age was 16 days, Majority of them (n=23, 58%) were severely cyanosed with SpO_2_ of 41.4±3.4% and underwent emergency BAS and remaining underwent elective procedure. An increase in SpO_2_% from 46.0±6% to 81.0±3.0% (p=<0.001) and ASD size from 1.4±2.8mm to 5.45±0.4mm was observed (p=<0.001). No complication was observed in most of cases (n=28, 70%). Mean hospital stay was 3.4±1 days. Success rate was 97.5% however, one neonate died due to neonatal sepsis.

**Conclusion:**

Our study provides sufficient evidence that bed side balloon atrial septostomy is a safer technique, save a lot of time and resources which were required otherwise in transporting these patients to catheterization laboratory.

## INTRODUCTION

D-loop transposition of the great arteries (d-TGA) is the second most common form of cyanotic congenital disease, with very high mortality (85-90%) if left untreated.^1^,^2^ The survival of these infants are entirely depends on adequate inter-circulatory mixing. This can be achieved by creating or widening of pre existing patent foramen ovale (PFO)/ Atrial septal defect (ASD) if adequate mixing is not present.^3^

First such procedure was surgically creation of ASD ‘Blalock–Hanlon Septectomy’ reported in 1950 considered as treatment of TGA.^4^ Despite of being a palliative procedure, this surgery was considered as landmark in surgical treatment of d-TGA. Later in 1966 Rashkind and Miller reported the percutaneous Balloon atrial septostomy in Catheterization laboratory.^5^ With the advent of ‘balloon atrial septostomy (BAS), the Blalock–Hanlon septectomy eventually was replaced.

BAS provides immediate relief by creating inter atrial mixing in neonatal and early infancy period but has few worrisome complications like balloon rupture, perforation of atrial appendage, and damage of pulmonary vein, inferior vena cava (IVC) and Mitral valve (MV).^6^

These complications remained worrisome until Matsunaga et al. tried to use echocardiography guided procedure which minimized most of the complications.^7^,^8^ In 1984 Baker et al.^9^ performed BAS in eight children with d-TGA only under echocardiography guidance; BAS monitored with echocardiography is a safe and effective procedure. It may be performed at bedside, avoiding transporting of the patient and radiation. Echocardiography helps in identifying catheter location and reduces the risk of perforations and lacerations of various structures, it also reduces the occurrence of other severe complications and immediate identification of these complications and echocardiography also assesses the immediate result of the procedure.^10^-^15^ In addition it reduces the burden of cardiac catheterization lab.

Pakistan is a developing and resource limited country with high birth rate (3.55 births per woman), low GDP and with limited physician to patient ratio (7.8/10000 population). There are nine paediatric cardiology centers in the entire country of more than 207 million. Paediatric cardiac services included cardiac catheterization laboratories are scares with limited skilled facilities. Emergency procedures including septostomy and use of catheterization unit is an extra burden on already over-burdened unit. Bed-side cardiac procedures require highly skilled and trained physician and staff with a back-up surgical unit support. The purpose of this study was to assess the effectiveness, safety, success and immediate complications of echocardiographic guided BAS in patients having d-TGA with intact ventricular septum in tertiary care hospital of Karachi, Pakistan.

## METHODS

### Study design and Population

We performed a prospective case series of forty infants at department of paediatric cardiology, National Institute of Cardiovascular Diseases (NICVD), Karachi, Pakistan from October 2016 to September 2017. NICVD was established in 1963, and is the largest public sector tertiary care cardiac center of the country provides services to more than 25000 children annually. Infants with echocardiographic findings consistent with d-TGA with IVS and restricted PFO of ≤2mm or small-size communication of less than 1/4^th^ of the length of the intact atrial septum (IAS) measured in the sub costal view (Fig.1a), without severe aortic, pulmonic, mitral or tricuspid stenosis or other significant cardiac or lung anomaly were included in this study after taken written informed consent from parents/guardians.

**Fig.1a:**
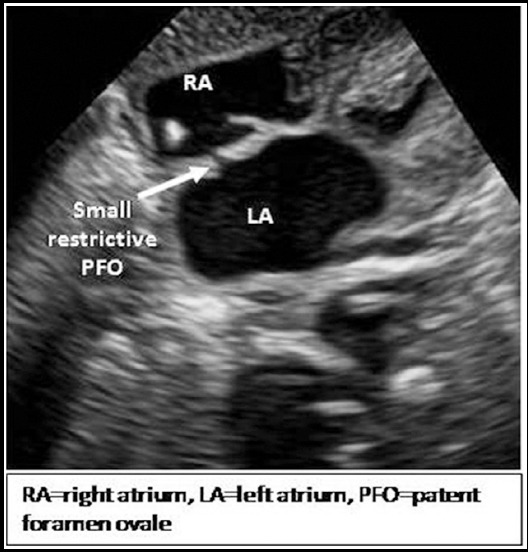
Sub costal four chamber view showing small restrictive PFO.

### Procedure technique

Echocardiography was performed continuously during procedure, standard echocardiographic windows and views were used, according to the recommendations of the American Society of Echocardiography^16^ and sequential segmental analysis was performed.^17^ The equipment used for the echocardiography assessment was (Toshiba Xarrio-200) with 12 MHz probe incorporating color, pulsed wave and continuous wave Doppler. Images were recorded as clip and still store. For BAS right or left femoral venous access was obtained by Seldinger technique. Guide wire’s position in inferior vena cava was confirmed by echocardiography in sub costal bicaval view (Fig.1b).^6^ French (Fr) introducer sheath was advanced over guide wire in femoral vein. 5Fr Miller BAS Catheter passed through venous sheath and advanced from IVC to right atrium (RA), course was monitored in sub costal sagital view. Catheter then advanced from right atrium (RA) to left atrium (LA) through PFO monitored in sub costal bicaval and four chamber views. After confirmation that balloon is away from LA appendage, mitral valve (MV) and pulmonary veins, it was inflated in the LA (Fig.1c) and withdrawn across IAS into the RA using a rapid and forceful jerk. Balloon was immediately deflated in mid of RA, the deflated catheter was advanced to the LA again and the procedure was repeated until adequate atrial communication or success criteria were achieved (Fig.1d). During procedure strict cardiac monitoring was done and any event like arrhythmias, apnea, fits, oxygen saturation, and hypotension was documented. After procedure, deflated balloon taken out of body and venous sheath was removed, dressing applied on puncture site as bleeding stopped from it. After procedure size of created ASD, peripheral SpO2 and number of tractions along with any possible cardiac complication were recorded. Difference in lower limbs temperature, color and bleeding from puncture site were monitored after procedure.

**Fig.1b:**
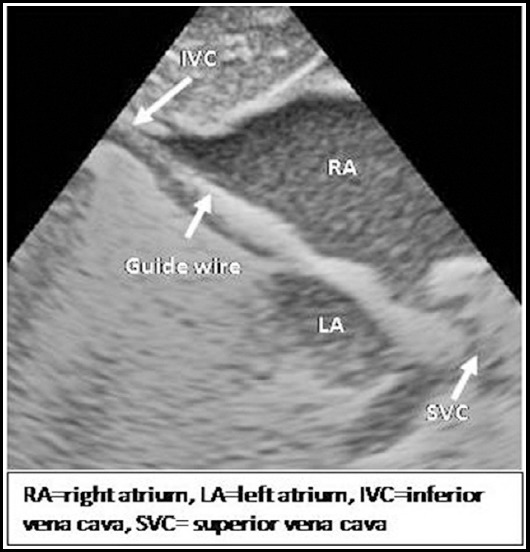
Sub costal bicaval view showing guide wire course from ICV to RA.

**Fig.1c:**
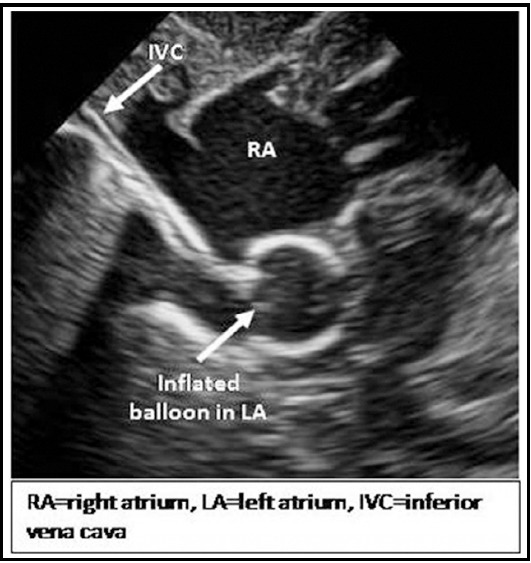
Sub costal modified four chamber view showing balloon catheter course from RA to LA.

**Fig.1d:**
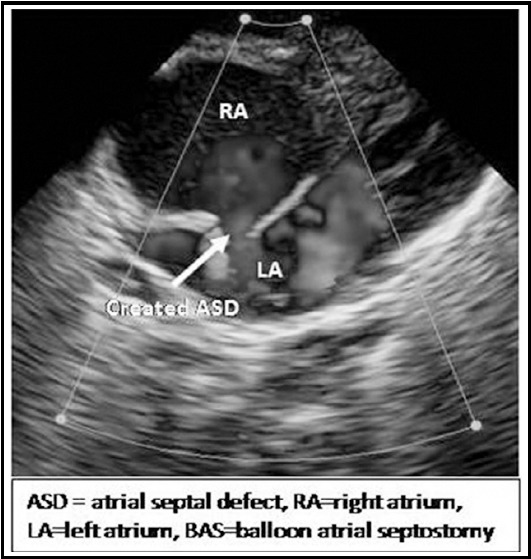
Sub costal modified four chamber view showing adequate sized created ASD after BAS.

### Definitions

### Procedure success

The *success criterion* of the procedure was the increase in the peripheral oxygen saturation, the increase in the atrial septal defect diameter > 1/3 of the total inter atrial septal diameter measured at the sub costal view or created ASD of around 5 mm with free margins mobility (Fig.1d) and clinical improvement.^10^,^13^,^18^

### Procedure related complication

Complications related to procedure may be Arrhythmia (bradycardia or tachycardia), apnea, balloon rupture; Femoral venous blockage, Venous congestion leads affected limb hypothermia and bluish discoloration.

### Data collection and analysis

Patient’s demographic data like age in day, gender, weight (kilograms), oxygen saturation (SpO_2_ %) at room air were recorded. Baseline blood tests as per hospital standard procedures (complete blood count, coagulation profile, C-reactive-protein, blood culture, Urea, Creatinine and chest X-Ray) were performed. Statistical package for social sciences (SPSS 21) was used for the analysis. Shapiro-Wilk (S-W) test was applied to check the hypothesis of normality, descriptive statistics such as mean ± SD, median (IQR), maximum and minimum were calculated for pre and post BAS diameter of ASD and oxygen saturation level. Paired sample t test was applied to analyze the effectiveness of bedside BAS. A two-sided p-value of ≤ 0.05 was taken as criteria for statistical significance.

## RESULTS

Out of 40 patients 22 (55%) were males and 18 (45%) were female. Median age of the patients was 16.5 (25.75) days. Mean weight of patients was 2.93 ± 0.30 kg. Eleven (28%) patients were on Prostaglandin E1 (PGE1).

Twenty three (58%) had severely cyanosis with SpO_2_ of 41±3.4 % and have emergency BAS, and done within 5.6±2.5 hours of admission. Remaining 17 (42%) had SpO_2_ of 51±3% so elective procedure was performed within 40.4±10 hours of admission after stabilization. Mean duration of procedure was 36±12 minutes.

**Fig.2:**
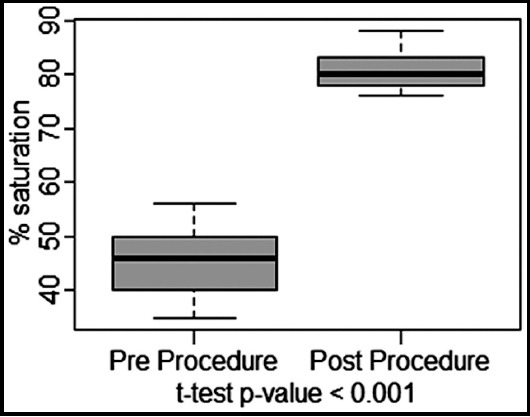
Oxygen Saturation level.

**Fig.3:**
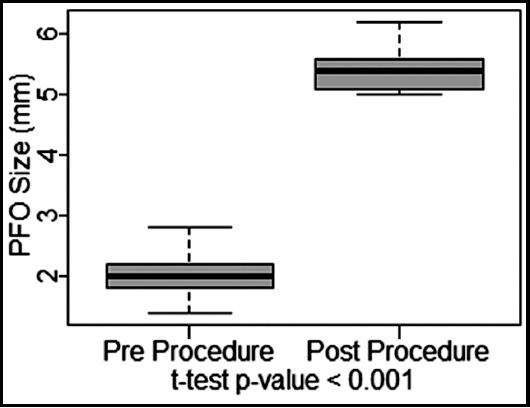
Created Atrial Septal Defect Size.

A significant increase of 35±7% in post procedure SpO2 % level form baseline (Fig.2), and 3.41±0.6mm of increase in post procedure Atrial Septal Size from baseline (Fig.3) were observed with p-value of <0.001 each. Immediate effect of BAS is presented in Table-I.

**Table-I:** Immediate effect of Balloon Atrial Septostomy (BAS).

	Mean±SD	Min-Max	Post Procedure Increment	P-value (t-test)
***Oxygen Saturation (%)***				
Pre Procedure	45.8±5.9%	35 - 56%	35.13±6.56%	<0.001*
Post Procedure	80.93±2.96%	76 - 88%		
***Atrial Septal Defect size (mm)***				
Pre Procedure	2.04±0.34mm	1.4 - 2.8mm	3.41±0.56mm	<0.001*
Post Procedure	5.45±0.39mm	5 - 6.2mm		

* Statistically significant at 0.05 level of significance.

**Fig.4:**
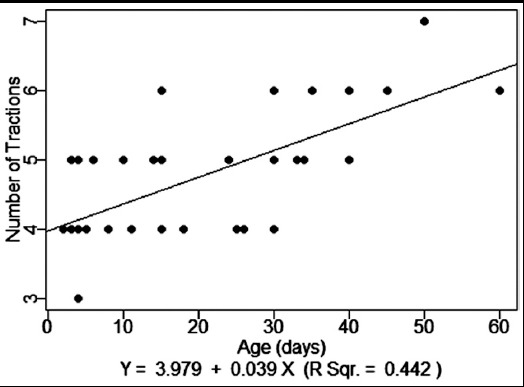
Age (days) vs. Number of Tractions.

Mean duration of hospital stay was 3±1 days, for 4 (10%) patients hospital stay was for 2 days, 3 days for 26 (65%) patients and for 10 (25%) patients hospital stay was more than 3 days. We have 97.5% success rate of BAS however 2.5 % (1) died unrelated to procedure due to neonatal sepsis. Twenty-seven (67.5%) patients were discharged to home and planned for Atrial Switch procedure (Senning Procedure) and 12 (30%) patients were referred for Arterial Switch Operation (ASO). Mean number of balloon tractions required were 5±1 (3-7). Required number of tractions were three in 1 (2.5%), four in 16 (40%), five in 14 (35%), six in 8 (20%), and seven in 1 (2.5%) of the patients. A scatter plot with fitted regression line between age of patient and number of tractions required is presented in Fig.4. A positive relationship was observed with R square of 0.442

Our safety profile was acceptable where Twenty eight (70%) patients were free of any complications, while remaining had following complication. Five (12.5%) had balloon rupture, (Out of these 3 patients were more than 30 days, one patient was 26 days and one was 15 days old). Fortunately no complication related to rubber fragments embolization seen in any of patient. Four (10%) went into apnea and bradycardia, recovered completely with short duration of cardiopulmonary resuscitation (CPR). Other insignificant complications includes Premature Atrial and Ventricular Contractions (APCs and PVCs respectively) in three (7.5%) patients, unfortunately one (2.5%) patient in our study aged 13 days died after the procedure who was severely cyanosed with O2 saturation of 30-35%, had frequent apnea episodes, required endotracheal intubation and Ventilatory support during procedure, however successful BAS was performed with size of created ASD of 5.5mm and O2 saturation increased to 85%, patient was on ventilator support and became septic, developed multiorgan dysfunction and died on third day of procedure.

## Discussion

Cyanotic congenital heart disease appears to be fatal in neonatal period especially if there is no inter atrial mixing like d-TGA, Total Anomalous Pulmonary Venous Connections (TAPVC), Tricuspid Atresia (TA), Mitral Atresia (MA). Creating or widening of inter atrial defect not only gives immediate physiological relief but also bridges the infant for surgical correction. Matsunaga et al.^8^ first reported the echo guided BAS technique which was later performed by Perry et al.^14^ in USA in 1981. Echocardiography guided bed side BAS has been proved to be a palliative, live saving, and emergency procedure with minimal side effects. We report our data of 40 patients with dTGA with IVS who had echo guided BAS. We have the same conclusion as Perry et al.^14^ that bed side echo guided BAS is life saving and less risky than conventional BAS done in cardiac catheterization laboratory, as location of catheter inside the heart can be identified accurately and helps in the assessment of defect size immediately. In 2005, Marchi CH et al.^11^ published their data with the mean age was 8.3±9.3 days and the median was 4 days, ranging from 1 to 46 days in contrast our data with the higher mean age of 21.3±15.22 median age of the study patients was 16.5 (25.75) days. It is due to delayed referrals which are due to of lack of health care facilities resulting in late diagnosis.

Marchi CH et al.^11^ reported mean weight of 3.1±1 kg (2.3- 6.5 kg) and the median was 3.3 kg, in contrast mean weight in our patient was 2.93±0.3kg (2.40 - 3.60 kg) and the median was 2.95kg. Study further reported the size of the created ASD increased from 1.8±0.8 mm to 5.8±1.3 mm and SpO2 % increased from 64.5±18.9% to 85.1±9.2%. Results of our study are comparable for both size of PFO/ASD and SpO2 % level with significantly increase of 2.04±0.34 pre-procedure to 5.45±0.39 post-procedure (p value <0.001) and 45.8±5.9% to 80.93±2.96% post-procedure (p value <0.001) respectively.

Matter M et al.^13^ published their data in 2011, they required an average of 5.23 tractions in order to get an effective defect which was relatively lesser in our data; while we required 4.80±0.88 balloon tractions. Most patients who required >4 balloon traction were more than 20 days old, however there was only one patient in whom seven tractions were needed and we failed to create significant communication in one or two attempts in any of patient. Numbers of balloon tractions were more in older infants. With age IAS become thick, less elastic and difficult to rupture to create adequate size ASD as compare to younger neonates in whom ASD was created easily with ≤4 tractions only. A moderate positive relationship between age of patients and number of traction was observed in our data with R-square of 0.442, on contrary Marchi CH et al.^11^ reported no correlation with R-square of 0.0285.

Finan E et al.^19^ proposed a late discontinuation of PGE1 in their work and showed a rebound hypoxemia after early discontinuation. We had only 11 (27.5%) patients with PGE1 administration; because most of our patients were elder and PDA was spontaneously closed in them, however, we try to discontinue PGE1 earlier after BAS and rebound hypoxemia was not observed in any of our patients. Nevertheless, it warrants further trials to observe the rebound hypoxemia after early discontinuation of PGE1. Twelve (30%) patients were referred for anatomical creation Arterial switch operation (ASO) in which LV was still able to support systemic circulation on echocardiography assessment i.e. LV Posterior wall Diameter (PWD) in end diastole >3.5mm, LV mass >35gm/m2 measured in parasternal long axis on M-mode, LV mass/Volume ratio >2 as good, 1.5 to 2 as acceptable, LV volume measured in apical four chamber view.

Twenty seven (68%) patients were not suitable for ASO because of Left Ventricle (LV) regression and its inability to support the systemic circulation to according to above mentioned criteria so were planned for physiological correction (Senning Procedure).

A cohort of dTGA was benefited because of this bed side procedure. Although no major complications were observed during these procedure and all possible safety measures were taken. Limited number of catheterization laboratories and centers are the factors leading to perform this bedside procedure. This is one center data and further local data is needed to strengthen literature.

## Conclusion

Our study provides sufficient evidence that bed side balloon atrial septostomy is a safer technique and save lot of time and resources which were required otherwise in transporting these patients to catheterization laboratory. Also bed side BAS avoids radiation exposure provides greater safety for positioning the balloon catheter, and immediate assessment of the result. Hence, bed side BAS is life and time saving, easier, cost effective and gives immediate result. BAS is very effective palliative procedure to stabilize sick patients before ASO/Senning Procedure in patients with dTGA.
